# An effective device to enable consistent scratches for in vitro scratch assays

**DOI:** 10.1186/s12896-023-00806-5

**Published:** 2023-08-28

**Authors:** Sixun Chen, Ahmad Amirul bin Abdul Rahim, Pamela Mok, Dan Liu

**Affiliations:** 1 Agency for Science, Technology and Research (A*STAR), Bioprocessing Technology Institute BTI, 20 Biopolis Way, Singapore, 138668 Singapore; 2 Celligenics Pte Ltd, 30 Biopolis Street, Singapore, 138671 Singapore

**Keywords:** Wounding healing, Scratch assay, Cell migration, Prototype, Device

## Abstract

**Background:**

The in-vitro scratch assay is a useful method in wound healing research to assess cell migration. In this assay, a scratch is created in a confluent cell layer by mechanically removing cells through manual scraping with a sharp-edged tool. This step is traditionally done with pipette tips and is unsuitable for high-throughput assays, as the created scratches are highly variable in width and position. Commercially available solutions are often expensive, and require specific cultureware which might not be suitable for all studies.

**Results:**

In this study, we have developed a flexible cell scratch device comprising a single wounding tool, a guide and an imaging template for consistent and reproducible scratch assays in 96-well plates. Our results showed that the device produced a more consistent scratch profile compared to the conventional method of using pipette tips. The imaging template also allowed operators to easily locate and image the same region of interest at different time points, which potentially could be used for other assays.

**Conclusions:**

Our flexible yet effective scratch device thus enables robust scratch assays that can be applied to different experimental needs, providing researchers with an easy and reliable tool for their studies.

## Background

The in vitro two-dimensional scratch assay on primary skin cells such as keratinocytes, endothelial cells, and fibroblasts is an important tool in preclinical wound research to understand the mechanism of actions regulating cell migration as well as the effects of cell migration under different experimental conditions [1, 2]. The assay provides a simple and economical way for a preliminary investigation on these mechanisms that can be further evaluated in more relevant complex in-vitro or in-vivo models.

The scratch assay involves culturing cells to confluence and creating a ‘scratch’ by removing a section of cells in the monolayer culture through mechanically scraping the surface with a sharp-edged tool [3–5]. This is typically performed by an operator scratching the cell layer using a pipette tip. Cells on each edge of the scratch would then be observed for migration towards the opposite edge until cell–cell contact is achieved, filling in the scratch. The scratch created in the cell monolayer is sometimes referred to as a ‘wound’, with the assay also known as a ‘wound healing’ assay [6]. Other methods are available for creating such ‘wounds’ in a cell layer [7] including stamping [8–10], electrical removing of cells [11] and enzymatic digestion (this method requires the culture of cells within a microfluidic device) [12–14] but the scratch assay is still widely used due to its low cost as it uses inexpensive and commonly found consumables in most laboratories. The pipette tip is commonly chosen as the sharp-edged tool in the assay as it is sterile, disposable, and widely available in different sizes for creating different-sized scratches.

Scratch assays with 96 wells enable high-throughput studies on the wound healing process under different experimental conditions [15]. This application requires consistent scratch widths and positioning across wells to ensure valid comparisons and reproducibility between assays [16]. Consistent scratch widths are, however, difficult to achieve with pipette tips due to factors such as variability in bore shape/size [17] and the force applied [18]. Although straight scratches can be performed with a simple guide such as a ruler, there will still be variability in scratch positioning [19], which complicates the process of fixing the well-to-well positioning for imaging. Consequently, scratching using pipette tips is not suitable for high-throughput scratch assays in 96-well plates.

Commercial solutions available for producing consistent scratches in 96-well scratch assays include Sartorius’s Incucyte Wound Maker and Agilent’s AccuWound 96, which require the Incucyte ImageLock 96-well plate and Agilent E-plate, respectively. The requirement of specific cultureware reduces the accessibility of these devices to studies that require the use of other cultureware. V&P Scientific Inc had a line-up of wounding pin tools for 96-well and 384-well assays which has since been discontinued (personal communication). However, their pin-transfer device is still available commercially and was used successfully in a study as a wounding tool in a scratch assay [20]. The aforementioned commercial solutions scratch all 96 wells in the plate simultaneously, which could limit experimental designs (e.g., inability to omit wells to be scratched or to study different scratch widths).

Tracking progress of cell migration and proliferation on wound edge can be performed using live cell imaging [20, 21] through templated cultureware in commercial solutions as described above or through creating reference points within or close to the scratch area using etchings of a razor blade or needle [5]. The Incucyte ImageLock 96-well plate, for example, has markers on the bottom of the plate to provide guides for accurate positioning of the plates while imaging. In particular, when pipette tips are used in scratch assays, the widths of the scratches are generally small, and cells have to be observed over short time intervals (every 0.5–3 h depending on cell line) to observe significant differences and acertain changes in migratory mechanics between controls and perturbed samples [22]. This leads to either the use of a live-cell imaging setup that is costly and not readily accessible or having to take images manually at all time points, which is time-consuming and laborious.

To address issues in manual scratching and commercial solutions, we developed a scratch device comprising a single wounding tool, a guide, and an imaging template for use in a 96-well scratch assay. We also report its development and demonstrate its effectiveness in comparison to scratches using pipette tips.

## Methods

### Design and fabrication of scratch device

**Fig. 1:**
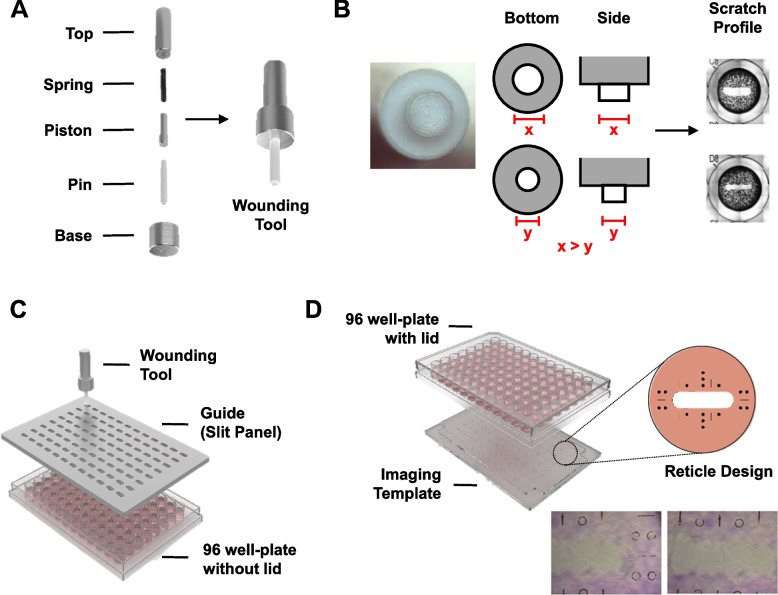
Design of Scratch Setup. **A** Parts of wounding tool consisting of the top, spring, piston, pin, and base. **B** Pin tip can be fabricated with different diameters resulting in different scratch widths profile. **C** Assembly of wounding tool through the guide (slit panel) which guides the scratch process within each well of the 96-well plate. **D** Assembly of the 96-well plate on the imaging template consisting of reticle design for the monitoring of cell migration over the scratch area

The scratch device comprises of a wounding tool, a guide and an imaging template (Fig. 1).

The design of the wounding tool (Fig. 1A) comprises a self-levelling pin using a spring. The pin is fabricated with polytetrafluoroethylene (PTFE) which is bioinert and biocompatible [23]. PTFE also has the lowest coefficient of friction compared to other available fabrication and bioinert plastics such as polycarbonate and polypropylene. This characteristic, combined with the tool’s self-levelling function, enables the PTFE pin to glide across the cell sheet, creating the gap while ensuring minimal to no deep surface scratch on the plate. In addition, PTFE is also naturally hydrophobic, enabling minimal cell adhesion to the material during the scratch process, allowing the pin to transfer from well to well with minimal to no cell and cellular components. The size of the pin tip can be fabricated into varying diameters to accommodate for different scratch widths required for the scratch assay (Fig. 1B). A stainless steel 304 (SUS304) spring of minimal allowable load ranging between 1.5–4.5 N is used to maintain a balance of force to ensure sufficient compression to create gaps within the cell sheet without damaging the plate substrate. The top, base, and piston are fabricated with stainless steel 316 (SUS316) which ensures that they remain robust and corrosion-resistant even with exposure to fluids and experimental conditions such as culture media, ionic buffers, 70% ethanol, autoclave conditions, and UV light. The assembled wounding tool can be sterilized by autoclave and used directly post-sterilization after drying.

The guide was designed as a slit panel (Fig. 1C) to guide the wounding tool through the scratch process. Flatness on the top and bottom of the slit panel was emphasized during fabrication to ensure parts in contact sits flatly with each other ― panel with the 96-well plate and wounding tool with the panel. This is important as it ensures that the wounding tool maintains its vertical orientation throughout the process and consequently that the pin tip is always in full contact with the bottom of the well. Per well, the slit has been designed to be positioned directly in the middle of the well and has a close running fit with the pin. Together, these factors enable the pin to glide smoothly in a controlled manner, with the scratch profile always in contact with the well bottom creating a straight gap in the cell sheet at the centre of the well that is consistent in width and angle across the well. Repeated consistent slits were designed following the dimensions of the 96-well plate. The panel is also fabricated in SUS316 as it is treated as a capital device that needs to be robust and able to withstand exposure to the same fluids and elements as the wounding tool.

The imaging template aids in monitoring cell migration across the gap created by the wounding tool. It was designed to be placed below the 96-well plate and to sit onto the inverted microscope stage with or without the manual x–y adjustment adaptor. The template was fabricated using clear poly(methyl methacrylate) (PMMA) with a Picosecond laser-etched reticle design (Fig. 1D). PMMA was selected because of its good optical and anti-scratch properties [24], which enables clear imaging and monitoring of cell migration, as well as ensuring that the panel is sufficiently robust for repeated mounting on and off the microscope and the 96-well plate. The thickness of the panel is approximately 5 mm, with clear top and bottom surfaces to enable unobstructed monitoring at 4 × magnification objectives on the inverted microscope. The top surface of the template has a sliding fit for ease of mounting and dismounting of the 96-well plate, as well as approximate placement of the laser-etched reticles. The reticle is positioned at the centre of the wells and consists of straight lines and circles that are approximately 500 µm apart. Picosecond laser with a resolution of 30 µm spot size was used to create the reticle design. The reticle patterns serve as reference points for the scratch boundary and are apparent when viewed under a phase contrast microscope.

### Cell culture

Human Dermal Fibroblast (HDF) (Lonza, Switzerland) cells were cultured on cultureware coated with 0.1% gelatin (PAN Biotech, Germany) in Dulbecco’s Modified Eagle’s Medium (DMEM) – low glucose (PAN Biotech, Germany) with 10% fetal bovine serum (FBS) (Biowest, France), 1% penicillin–streptomycin (10,000 U/mL) (Gibco, USA), 1% GlutaMAX™ (100 ×) (Gibco, USA), 1% MEM non-essential amino acids solution (100 ×) (Gibco, USA) and 50 mM 2-Phospho-L-ascorbic acid trisodium salt (MilliporeSigma, USA).

HaCaT cells (AddexBio, USA) were cultured on cultureware coated with 0.1% gelatin (PAN Biotech, Germany) in DMEM (Gibco, USA) with 10% FBS (Biowest, France), 1% penicillin–streptomycin (10,000 U/mL) (Gibco, USA), 1% GlutaMAX™ (100 ×) (Gibco, USA) and 1% MEM non-essential amino acids solution (100 ×) (Gibco, USA).

### Scratch assay

HDF cells (4,000 cells/well) and HaCaT cells (10,000 cells/well) were seeded in 96-well plates (Corning, USA) in complete media and incubated at 37 °C and 5% CO_2_ for 48 h. Scratches by wounding tool: The wounding tool and guide were sterilised with 70% ethanol and placed in the biosafety cabinet (BSC). The cover of the 96-well plate was replaced by the guide and the wounding tool was inserted into the slit of the guide, pushed down, and slid along the slit to create a scratch on the cell surface. The motion was repeated for wells scratched by wounding tool. Scratches by pipette tip: The cover of the 96-well plate was used as a straight-edged guide. Using a pipette tip, scrape the cell surface in a straight line guided by the cover. The motion was repeated for wells scratched by pipette tip. Cells were then fixed with 2% paraformaldehyde (Thermo Fisher Scientific, USA) and stained with 0.25% Safranin O (EMD Millipore Corp, USA). The plate was then mounted onto the imaging template and placed on the stage of an Olympus IX71 inverted microscope. 4 × phase-contrast and brightfield images were taken which were then analysed using Image J (https://imagej.net/ij/index.html).

### Statistical analysis

Variance in scratch widths between scratches generated by different scratch tools (Device vs 200 uL tip, Device vs 1000 uL tip) were analysed using two-sample F-tests for variances. All calculations were computed using Microsoft Excel (Office 365). Data are expressed as mean ± standard deviation.

## Results

### Device design

With the considerations described above, the finalized design of the wounding tool (Fig. 1A) comprises a self-levelling pin using a spring. We observed that this property enabled the wounding tool to create consistent gaps in a confluent cell layer across multiple wells of a 96-well plate. In addition, this ensured that there were no deep scratches on the growth surface that could affect cell migration. Our observations were consistent across different users and well depth variability (data not shown).

### Device produced more consistent scratch widths and positions compared to pipette tip

**Fig. 2:**
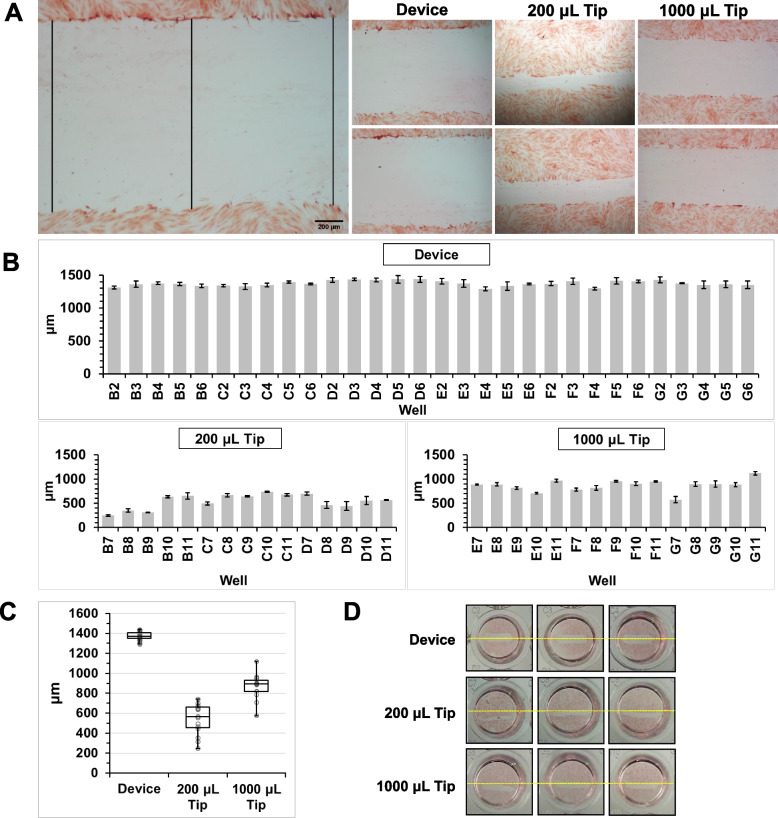
Scratch assay on HDF cell line. **A** Representative images of scratch profiles created by the scratch device (wounding tool and guide), 200 µL tip and 1000 µL tip. Black lines indicate sites where measurements were taken for analysis of scratch widths. **B** Scratch width measurements. Standard deviations were calculated from measurements across the scratch as shown in **A**. **C** Box and whisker plot indicating minimum, maximum, median, upper, and lower quartile values across the means of all wells. **D** Representative whole well image of scratches. Yellow lines go through the centre of the wells

To compare the performance of the device against pipette tips (200 µL and 1000 µL), scratches were performed on primary human dermal fibroblasts (HDF) (200 µL and 1000 µL tip) and immortalised human keratinocyte (HaCaT) (1000 µL tip) cell lines. For this experiment, pins with diameters of 1.7 mm were carefully fabricated and inspected through an external micrometer to ensure that the specified tolerances were achieved (measured: 1.696–1.699 mm). Scratches were first made with the wounding tool followed by the pipette tips across each row sequentially (wounding tool: columns 2–6, pipette tips: columns 7–11). Cells were fixed and stained after scratching. Images of each well were taken at 4 × magnification and scratch widths evaluated for their positioning and consistency. Scratch widths were measured across three gaps in each image (Fig. 2A).

HDF cultures were scratched using the wounding tool with the guide (30 wells), and the 200 µL and 1000 µL pipette tips using the edge of the plate cover as a ruler (15 wells each). The wounding tool generated scratches with larger widths and smaller deviations (1373.9 ± 52.2 µm) compared to the 200 µL tips (543.0 ± 153.0 µm) and 1000 µL tips (870.8 ± 126.7 µm) (Fig. 2A–C). An F-test was performed to assess any significant differences between the wells within each treatment. The results showed that the variance between the scratches created by the wounding tool (σ^2^_tool_ = 1737) was significantly lower than those created by the 1000 µL tips (σ^2^_1000μL_ = 23037) (F_1,43_ = 13.26, *p* < 0.01) and 200 µL tips (σ^2^_200μL_ = 15487) (F_1,43_ = 8.92, *p* < 0.01). The use of the guide with the wounding tool created uniform scratches close to the centre of the wells compared to scratches created by pipette tips (Fig. 2D). The wounding tool was able to generate these scratches more consistently with lower intra-well and inter-well variability.

**Fig. 3:**
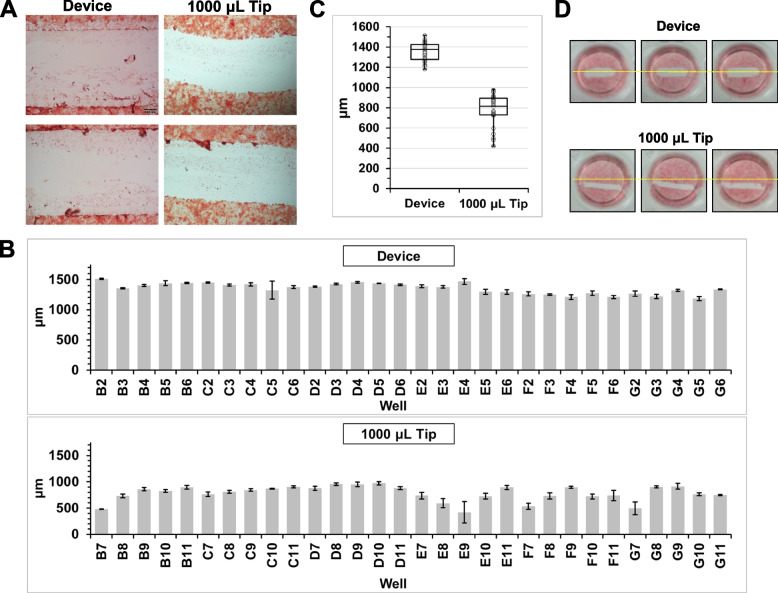
Scratch assay on HaCaT cell line. **A** Representative images of scratch profiles created by the scratch device (wounding tool and guide) and 1000 µL tip. **B** Scratch width measurements. Standard deviations were calculated from measurements across the scratch, as shown in Fig. 2A. **C** Box and whisker plot indicating minimum, maximum, median, upper, and lower quartile values across means of all wells. **D** Representative whole well image of scratches. Yellow lines go through the centre of the wells

The wounding tool was also compared with the use of pipette tips in generating scratches in HaCaT cell lines. As the 200 µL tips were generating much smaller scratch widths than desired in the HDF assay (Fig. 2), only 1000 µL tips were used in this evaluation with HaCaT cells. Both the wounding tool and 1000 µL pipette tip were used to scratch 30 wells each. Similar to HDF culture, the wounding tool generated larger scratch widths (1354.3 ± 90.0 µm) compared to 1000 µL tips (781.8 ± 148.6 µm) with lower intra-well and inter-well variability (Fig. 3A–C). The variance between scratches created by the wounding tool (σ^2^_tool_ = 8106) was significantly lower than those created by the 1000 µL tips (σ^2^_1000μL_ = 22076) (F_1,58_ = 2.72, *p* < 0.01). The widths of the scratches generated with the wounding tool were smaller from E5 to G6 compared to earlier scratched wells of B2–E4 which could be due to accumulated cell debris on the pin (Fig. 3B). The use of the guide with the wounding tool in the HaCaT culture also created scratches close to the centre of the wells compared to the pipette tip (Fig. 3D).

**Fig. 4:**
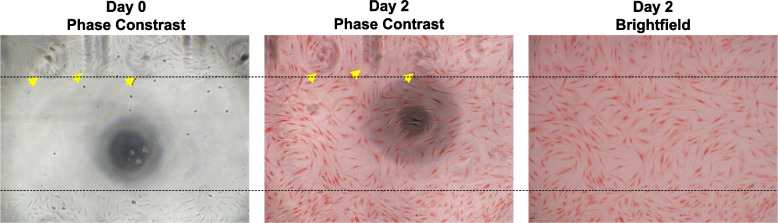
Use of the imaging template. Representative images of the wells with imaging template marking shown (yellow arrows) on Day 0 and Day 2. Black dotted lines indicate scratch borders on Day 0

The use of the imaging template was also evaluated in a regular scratch assay where cells were cultured for two days after the scratch was performed to allow for cell migration over the scratch width. In this case, the scratch was imaged with the template using phase contrast to note the initial scratch width and template marking positions on Day 0 (Fig. 4). After two days in culture, the cells were fixed, stained, and imaged again on Day 2. The template markings taken on Day 0 can be easily located using the phase contrast setting and is not visible using the brightfield setting of the light microscope (Fig. 4).

## Discussion

This study aimed to develop a simple wounding tool for a high throughput 96-well scratch assay. From our experience, the conventional method of using a 200 uL pipette tip in a 96-well plate did not yield consistent scratches. In addition, etching the bottom of each well with a needle (with the aid of the microscope) as positional reference points was tedious and time-consuming. Despite utilizing a ruler or edge of the plate cover as a guide to position the pipette tip to create straight scratches at the desired position within each well, scratches were still highly variable between wells and rendered data analysis almost impossible. To address the various challenges we faced, we designed and fabricated a scratch device to have these three major components—a wounding tool for removing the cells without creating deep scratches on the surface of cultureware, a guide to position the wounding tool consistently, and an imaging template to enable microscopic imaging of the cells in the same field over time. We then validated its utility for producing consistent and robust scratches for assays and demonstrated its improved performance over the conventional method of manual scratching using a pipette tip.

During the design of the wounding tool and guide, considerations were made to ensure maintenance of sterility while creating a clean gap with straight parallel edges and with minimal width variation within and between wells to enable valid tracking of cell migration and comparison between wells. These considerations focused on function, consistency, and compatibility with cells in culture. Specifically, we focused on factors that could cause variations, such as user handling variability, well dimension differences (primarily the z-axis height), durability and prototyping limitations. In addition, the design should ensure minimal to no deep scratches on the plate while performing the scratch, which could affect the cell migration process. Materials for the wounding tool and guide were chosen based on their biocompatibility and compatibility with sterilization methods such as autoclave and ethanol wipes, therefore most parts were fabricated using stainless steel due to its durability of wear. Consequently, PTFE was chosen as the most suitable material for the pin portion of the wounding tool, although it could eventually wear off after multiple use. Therefore, the pin was designed as a consumable that could be easily replaced.

A different set of considerations were taken when designing the imaging template. Its design focuses on the imaging process to track cell migration over the scratch width quickly without the use of an expensive live cell imaging setup or manual etching of each well. Therefore, factors affecting the imaging clarity of the reticle and cells superimposed on each other were examined. As the template is not used under aseptic conditions, a wider variety of materials were available as sterility and biocompatibility was not required.

With the considerations and materials chosen above, multiple iterations of the wounding tool and guide were tested prior to finalizing the version used in the present study. The differences in the iterations stem from the precision in design and fabrication of the parts as both the guide and pin control the width, position, and profile of the scratch. At the microscopic level, tiny changes in tool size or hand movements while scratching are magnified and can lead to poor consistency and large variations in the profile and position of the created scratches. One iteration included a multi-well row-by-row scratch setup that highlighted to us the minute differences in well depths within a plate and importance of consistent force application in each scratch. The dimensions and profile of the guide and pin, and the forces of the springs were tested and optimized to achieve consistent and user-independent scratch profiles in the current design. For future iterations, the performance of the wounding tool can be further improved by switching out the current method of pin fabrication, CNC machining, for molding. We have observed that CNC machining of the pin from a PTFE block caused high heat transfer during the process, leading to warpage of the pin hence lowering the uniformity of pins produced. The molding method enables both the pin and the piston to be fabricated as a singular unit allowing for minimal warpage, dimensional consistency, as well as smoother pin-piston compression and decompression. The consistency in the movement and dimensions achieved would then enable the tightening of the tolerances on the guide (slit panel) and the internal dimensions of the housing (top and base). All in all, the fabrication of the pin-piston unit through molding leads to the minimization of deviations in the scratch movement resulting in tighter and more accurate scratch widths. In addition, having a singular pin-piston unit improves user experience by making the replacement of the worn pin easier as it eliminates the need to remove and install the pin into the stainless-steel piston as seen in the current design. Although molding of pin-piston unit produces a more consistent wounding tool, it is not a cost-effective method and should only be considered for mass production.

In addition to the wounding tool and slit panel, multiple iterations of printing and etching methods of the reticle pattern were also tested on the PMMA panel of the imaging template to achieve line widths that minimizes disruption in the imaging process. These iterations included laser printing on a transparency sheet pasted over the panel, CNC engraving with a 0.3 mm flat end mill, and a CO_2_ laser engraving on the panel. However, the transparent sheet introduced multiple artifacts under magnification and the resolutions achieved by CNC and CO_2_ laser engraving (> 30 um) were deemed insufficient. The final design of the imaging template was fabricated with picosecond laser with a spot size of 30 um which ws able to produce etchings that were subtle enough to appear in view where the cells are in focus, yet not interfere with imaging process. It is also possible to achieve finer resolution using a UV laser with a finer spot size and this method can be explored for use in the future iterations of the imaging template.

From the efforts put into optimization of the current design, we observed that minute changes such as using pins with difference of 0.2 mm in diameter (1.5 mm and 1.7 mm) in the HaCaT culture produced magnified changes in the scratch widths (650 μm and 1500 μm respectively). This observation together with others during the development led us to conclude that the high precision required in fabrication of the wounding device likely poses a challenge in the manufacturing of the parts, which may account for the lack of such tools in the market and limitations in existing tools.

The finalised device described in this study uses a wounding tool with a single pin that scratches wells individually with a guide. This setup allows for flexibility in designing experiments that may not require all the wells within the 96-well plates—a feature that is not possible in commercially available tools that scratches all 96 wells at a time. It is also possible to fabricate pins with different tip diameters to allow for varying scratch widths as mentioned above in our experience using 1.5 mm and 1.7 mm pins. As the scratch widths directly correlate with the time points for observing wound closure and the mechanics of the closure is not linear, having the option to vary scratch widths for one’s assays could be useful in studying factors affecting extended migration in wounds [25, 26]. Although the cell lines used in this assay resulted in similar scratch widths, it may not be the case for all cell lines. Pins with different diameters may have to be tested and optimised for use on different cell lines. We observed a decrease in scratch widths over time when scratching HaCaT, which was not observed for HDF samples (Fig. 3B). This might be due to HaCaT cultures having smaller cell sizes and higher cell numbers, thereby generating more debris when the scratch was created. The increase in debris load might lead to the accumulated debris on the pin and create inefficient scratches. Frequent cleaning of the pin with 70% ethanol wipes between scratches could mitigate this issue. Furthermore, the guide can also be modified for different plate types, such as a 24-well plate, or for different scratch positioning. The imaging template can also be modified to complement these changes. Taken together, the ability to modify the parts makes the setup adaptable to a wide variety of studies which has not been highlighted in other scratch assay devices published for 96-well plates [27, 28].

The improved performance of our wounding tool over conventional pipette tips can be attributed to the accurate positioning of the tool using the guide and consistent scratching force in a fixed perpendicular downward direction over the scratch area. These features are also found in commercial wounding tools such as Sartorius’s Incucyte Wound Maker and Agilent’s AccuWound 96. It is noted that Incucyte Wound Maker and AccuWound 96 can create uniform scratches across all 96 wells simultaneously, making them more efficient compared to both pipette tips and our wounding tool. However, they come in fixed scratch widths/positions, require specialized plates, and may involve high-cost imaging systems in the case of Incucyte [29]. These limitations might not be suitable for some studies, highlighting a need for an alternative and more flexible wounding tool. There have been device designs and prototypes published improving on the manual scratching method in a 96-well plate such as an 8-channel device utilizing pipette tips developed by Poon et al. [28] and 96-well wound making pin setup developed by Lee et al. [27]. Another interesting concept for scratch device was developed by Fenu et al. using magnet guiderails to drag magnet placed into the culture, creating a wound [30]. This method however was developed using a 12-well plate and scaling down to a 96-well plate might prove difficult due to the requirement of manual placement of magnets into the culture. The limited device development studies published for scratch assays in a 96-well plate could be due to the difficulty in achieving the resolution and precision required at this scale. Our device design included novel concepts (imaging template, varying tip sizes, varying guides) and we believe that the flexibility and reliability of our wounding tool in creating consistent scratches, together with its low cost, make it a viable alternative to existing commercial methods and other published studies.

## Conclusion

We developed an effective scratch device to enable consistent and reproducible in-vitro scratch assays. Our tests demonstrated its superior performance in creating scratches of consistent and repeatable widths and positions in 96-well plates compared to using a pipette tip-based method. We have also verified its ease of use for microscopic imaging of cell migration compared to traditional laboratory means. Our device provides a simple, flexible, and useful tool to help researchers perform high quality scratch assays for wound healing studies and cell biology research.

## Data Availability

The datasets generated during and/or analysed during the current study are available from the corresponding author on reasonable request.
